# Expanded endothelial progenitor cells mitigate lung injury in septic mice

**DOI:** 10.1186/s13287-015-0226-7

**Published:** 2015-11-26

**Authors:** Andreas Güldner, Tatiana Maron-Gutierrez, Soraia Carvalho Abreu, Debora Gonçalves Xisto, Alexandra Cristina Senegaglia, Patty Rose da Silva Barcelos, Johnatas Dutra Silva, Paulo Brofman, Marcelo Gama de Abreu, Patricia Rieken Macedo Rocco

**Affiliations:** Department of Anesthesiology and Intensive Care Medicine, University Hospital Dresden, Technische Universität Dresden, Dresden, Germany; Laboratory of Pulmonary Investigation, Health Sciences Center, Carlos Chagas Filho Biophysics Institute, Universidade Federal do Rio de Janeiro, Ilha do Fundão, Rio de Janeiro, Brazil; Laboratory of Immunopharmacology, Instituto Oswaldo Cruz, Fundação Oswaldo Cruz, Rio de Janeiro, Brazil; Center for Cell Technology, Pontifícia Universidade Católica do Paraná, Curitiba, Paraná Brazil

**Keywords:** Sepsis, Inflammation, Growth factors, Cell therapies

## Abstract

Endothelial progenitor cells (EPCs) improve survival and reduce organ failure in cecal ligation and puncture-induced sepsis; however, expanded EPCs may represent an even better approach for vascular repair. To date, no study has compared the effects of non-expanded EPCs (EPC-NEXP) with those of expanded EPCs (EPC-EXP) and mesenchymal stromal cells of human (MSC-HUMAN) and mouse (MSC-MICE) origin in experimental sepsis. One day after cecal ligation and puncture sepsis induction, BALB/c mice were randomized to receive saline, EPC-EXP, EPC-NEXP, MSC-HUMAN or MSC-MICE (1 × 10^5^) intravenously. EPC-EXP, EPC-NEXP, MSC-HUMAN, and MSC-MICE displayed differences in phenotypic characterization. On days 1 and 3, cecal ligation and puncture mice showed decreased survival rate, and increased elastance, diffuse alveolar damage, and levels of interleukin (IL)-1β, IL-6, IL-10, tumor necrosis factor-α, vascular endothelial growth factor, and platelet-derived growth factor in lung tissue. EPC-EXP and MSC-HUMAN had reduced elastance, diffuse alveolar damage, and platelet-derived growth factor compared to no-cell treatment. Tumor necrosis factor-α levels decreased in the EPC-EXP, MSC-HUMAN, and MSC-MICE groups. IL-1β levels decreased in the EPC-EXP group, while IL-10 decreased in the MSC-MICE. IL-6 levels decreased both in the EPC-EXP and MSC-MICE groups. Vascular endothelial growth factor levels were reduced regardless of therapy. In conclusion, EPC-EXP and MSC-HUMAN yielded better lung function and reduced histologic damage in septic mice.

**Electronic supplementary material:**

The online version of this article (doi:10.1186/s13287-015-0226-7) contains supplementary material, which is available to authorized users.

## Findings

### Background

Mesenchymal stem cell (MSC) treatment has been widely used in many experimental models including sepsis [1–6]. MSC mechanisms of action might include: a decrease in alveolar–capillary barrier permeability [4, 6, 7]; enhanced alveolar fluid clearance [8, 9]; a shift in macrophage profile from pro-inflammatory to anti-inflammatory [10]; improved bacterial clearance [11, 12]; and mitochondrial transfer [13].

Sepsis leads to several immunological events that alter endothelial function in the macrocirculation and microcirculation [14]. Endothelial barrier dysfunction and microvascular leak contribute critically to the pathogenesis of organ failure in sepsis and of sepsis-related complications such as acute respiratory distress syndrome (ARDS) [15]. Therefore, reconstitution of the endothelial layer might be initiated via migration and proliferation of surrounding mature endothelial cells (ECs). Since differentiated ECs have a low proliferative potential, their capability to substitute damaged endothelium is restricted. Studies have observed that endothelial progenitor cells (EPCs) are increasingly mobilized during sepsis and that their migration is associated with clinical outcome [16, 17]. EPCs are precursor cells that can differentiate into mature ECs and create new blood vessels [18]. Generally, EPCs can be identified on the basis of their expression of CD133, CD34, KDR, and/or VE-cadherin cellular markers [19]. A recent study reported that EPCs improve survival and reduce organ failure in experimental sepsis [20]. Nevertheless, expanded EPCs represent an even better approach for vascular repair in myocardial ischemia [20, 21]. To date, no study has compared the effects of non-expanded EPCs (EPC-NEXP), expanded EPCs (EPC-EXP), and MSCs of human (MSC-HUMAN) and mouse (MSC-MICE) origin in experimental sepsis.

Within this context, we hypothesized that human umbilical cord blood-derived EPCs would be non-inferior or superior to MSCs at improving survival, lung function, and histology in experimental sepsis. The aim of the present study was to compare the efficacy of expanded and non-expanded human EPCs and human or murine MSC therapy in treating lung injury in a murine model of sepsis.

## Methods

This study was approved by the Ethics Committee of the Health Sciences Centre, Federal University of Rio de Janeiro (CEUA-019). All animals received humane care in compliance with the “Principles of Laboratory Animal Care” formulated by the National Society for Medical Research and the “Guide for the Care and Use of Laboratory Animals” prepared by the National Academy of Sciences, USA.

### Isolation and expansion of EPCs

Mononuclear cells were isolated from human umbilical cord blood. EPCs (CD133+) were selected using CD133-coupled magnetic microbeads (Miltenyi Biotech, Bergisch Gladbach, Germany), following the manufacturer’s instructions. After isolation, CD133+ cells were expanded as described elsewhere [22]. Immunophenotypic analysis was performed by staining 5 × 10^5^ isolated and expanded EPCs. EPCs were analyzed after isolation, whereas expanded cells were analyzed after 30 days of culture. The cells were incubated with various conjugated monoclonal antibodies against the following human antigens: CD133; CD34; CD45; CD14; CD31; CD105; and von Willebrand factor. Quantitative analyses were performed using a FACSCalibur flow cytometer and FlowJo software (Flowjo, Ashland, OR, USA) [22].

### Isolation and expansion of human MSCs

Bone marrow-derived human MSCs were isolated and expanded as described elsewhere [23].

Immunophenotypic analysis was performed by staining 5 × 10^5^ expanded human MSCs. The cells were incubated with conjugated monoclonal antibodies against the following human antigens: CD45; CD14; CD 90; CD73; CD166; CD105; HLA-DR; CD34; CD29; and CD19. Quantitative analyses were performed using a FACSCalibur flow cytometer and FlowJo software (Flowjo) [22].

### Isolation and expansion of mouse MSCs

MSCs from mice were isolated from 8-week-old BALB/c mouse femur and tibia bone marrow stromal cells as described elsewhere [2]. Immunophenotypic analysis was performed by staining 1 × 10^5^ expanded mouse MSCs. The cells were incubated with conjugated monoclonal antibodies against the following antigens: CD19; CD34; CD45; CD29; and Sca1+. Quantitative analyses were performed using a FACSCalibur flow cytometer and FlowJo software (Flowjo).

### Experimental protocol

BALB/c mice (8–10 weeks old, n = 169) were used. Sepsis was induced by cecal ligation and puncture (CLP) on day 0 [3]. Briefly, animals were anesthetized with sevoflurane and a midline laparotomy was performed. The cecum was carefully isolated and a 3–0 cotton ligature was placed below the ileocecal valve to prevent bowel obstruction. Finally, the cecum was punctured twice with an 18-gauge needle. In the sham group, an abdominal incision was made, but there was no cecal ligation or perforation. Both layers of the abdominal cavity were closed, followed by fluid resuscitation (sterile saline) subcutaneously. Sham and CLP animals received tramadol (0.05 mg/kg body weight, subcutaneously) for postoperative analgesia, repeated every 8 hours. After this step, animals were returned to their cages, where they received water and food ad libitum.

On day 1, the mortality rate in the CLP group was 34 %. The surviving animals were randomized to be euthanized for evaluation of lung mechanics, histology, and inflammatory mediators in lung tissue, or treated with the following therapies: saline (0.05 ml), EPC-EXP, EPC-NEXP, MSC-HUMAN, or MSC-MICE (1 × 10^5^ in 0.05 ml saline, intravenously), after which animals were analyzed on day 3. EPC-NEXP were extracted and injected, whereas EPC-EXP, MSC-HUMAN and MSC-MICE were used at the third passage. To evaluate the cell viability, cells were subjected to trypan blue exclusion assay. For trypan blue staining, cell suspension was mixed with 0.4 % trypan blue solution at a 1:1 ratio. After 1–2 minutes incubation at room temperature, the mixture was loaded onto one chamber of a Neubauer hemocytometer and squares of the chamber were observed under a light microscope. The viable/live (clear) and non-viable/dead (blue) cells were evaluated. The number of viable cells was calculated using the formula: (number of live cells counted/total number of cells counted) × 100. From the number of viable cells, we calculated the exact concentration to obtain 1 × 10^5^ cells in 0.05 ml.

Lungs mechanics, histological data and mediators in lung tissue homogenate (n = 8 for each experimental group) were measured, as described in the following sections.

### Lung mechanics

On days 1 and 3 after induction of sepsis, mice were sedated (diazepam 1 mg, intraperitoneally), anesthetized (thiopental sodium 20 mg/kg, intraperitoneally), tracheotomized, paralyzed (vecuronium bromide 0.005 mg/kg, intravenously), and mechanically ventilated. The anterior chest wall was surgically removed and a positive end-expiratory pressure of 2 cmH_2_O was applied. After a 10-minute ventilation period, lung static elastance (Est,L) was measured by the end-inflation occlusion method [24].

### Lung histology

The left lung was fixed in 4 % buffered formaldehyde solution, paraffin-embedded, cut into slices (4 μm thick), and stained with hematoxylin and eosin. Diffuse alveolar damage (DAD) was quantified using a weighted scoring system. In brief, values from 0 to 4 were used to represent the severity of edema, inflammation and atelectasis, with 0 standing for no effect and 4 for maximum severity. In addition, the extent of each score characteristic per field of view was graded on a scale of 0 to 4, with 0 standing for no visible damage and 4 for complete involvement. Scores were calculated as the product of severity and extent of each feature, and ranged from 0 to 16. Finally, the overall DAD score was calculated as the sum of single score characteristics, yielding values from 0 to 48 [25].

### Protein expression of inflammatory mediators and growth factors

Protein expression of interleukin (IL)-1β, IL-6, IL-10, tumor necrosis factor (TNF)-α, vascular endothelial growth factor (VEGF), and platelet-derived growth factor (PDGF) was measured in the lung tissue of EPC-NEXP, EPC-EXP, MSC-HUMAN, and MSC-MICE animals using commercially available enzyme-linked immunosorbent assay kits, in accordance with manufacturer instructions.

### Statistical analysis

Functional variables were tested with one-way analysis of variance followed by Tukey’s post-hoc test (Prism for Mac, Version 5.0a, GraphPad Software). The Kruskal-Wallis test followed by Dunn’s post-hoc test was used to compare DAD scores and molecular biology data. Survival rates were compared by log-rank test. Data are expressed as mean ± standard deviation or as median and interquartile range as appropriate. Significance was accepted at *P <* 0.05.

## Results

### Phenotypic characterization

The staining patterns of cells are provided in Table 1.

**Table 1:** Phenotypic characterization

	EPC-NEXP	EPC-EXP	MSC-HUMAN	MSC-MICE
CD45	3.52 %	1.54 %	0.25 %	0.32 %
CD34	68.6 %	22.5 %	0.17 %	0.24 %
CD14	–	0.48 %	1.79 %	–
CD133	94.9 %	23.0 %	–	–
CD105	–	99.6 %	82.0 %	–
CD73	–	–	84.4 %	–
CD29	–	96.8 %	86.2 %	99.1 %
CD90	–	–	92.6 %	–
CD166	–	87.9 %	63.4 %	–
CD19	–	–	0.17 %	0.71 %
CD31	–	82.6 %	–	–
HLA-DR	–	–	0.98 %	–
CD146	–	95.1	–	–
Sca-1	–	–	–	88.6 %
vWF	–	97.5 %	–	–

*EPC-CD* Cluster of differentiation, *EXP* expanded endothelial progenitor cell, *EPC-NEXP* non-expanded endothelial progenitor cell, *HLA* Human leukocyte antigen, *MSC-HUMAN* Mesenchymal stem cell of human origin, *MSC-MICE* Mesenchymal stem cell of mouse origin, *Sca-1* Stem cell antigen-1, *vWF* Von Willebrand factor

### Survival rate

The survival rate of untreated animals (CLP) was 66 % on day 1 and 59 % on day 3 (out of 100 % on day 0). On day 3, the survival percentage did not differ among untreated CLP animals, and the MSC-MICE, MSC-HUMAN, EPC-NEXP, and EPC-EXP groups (89, 96, 82, 76, and 100 % respectively). These percentages were calculated from the CLP animals that had survived through day 1 (See Additional file 1).

### Expanded EPCs and human MSCs ameliorated lung mechanics

**Fig. 1:**
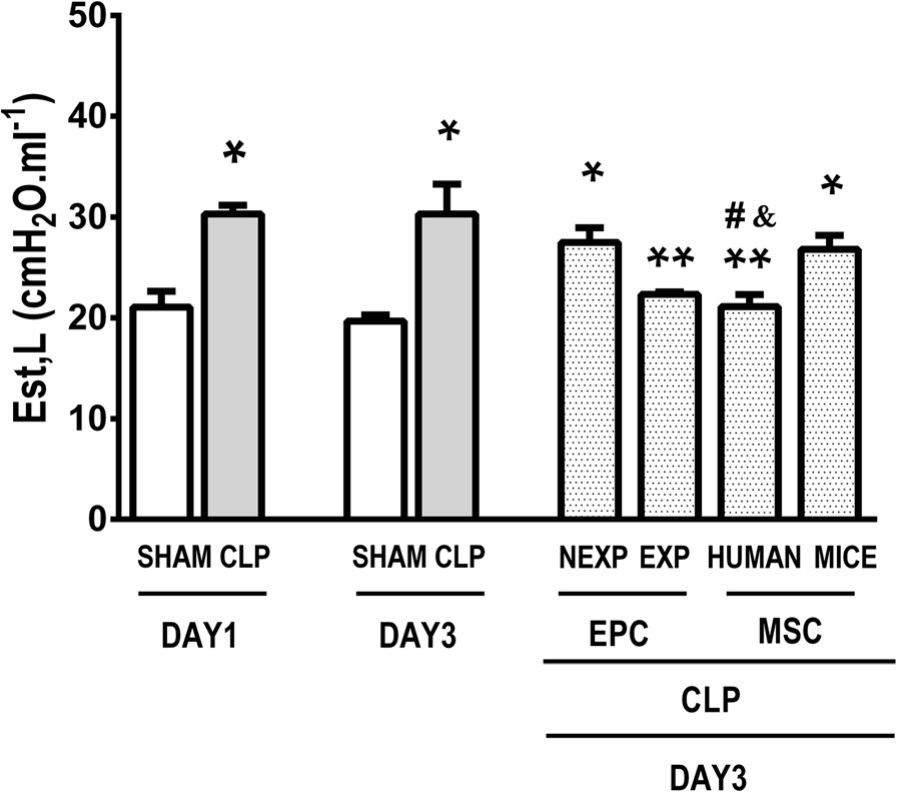
Static lung elastance on days 1 and 3. Mice were subjected to cecal ligation and puncture (*CLP*). A sham-operated group was used as a control CLP. At day 1, some animals were euthanized after establishment of ARDS in order to evaluate static lung elastance (*Est,L*), while other animals were randomized to receive saline or non-expanded endothelial progenitor cells (*EPC-NEXP*), expanded endothelial progenitor cells (*EPC-EXP*), human mesenchymal stem cells (*MSC-HUMAN*), or mouse mesenchymal stem cells (*MSC-MICE*), intravenously. Values expressed as mean ± standard deviation of eight animals in each group. *P < 0.05, versus respective sham group. **P < 0.05, versus CLP group at day 3. ^#^ *P <* 0.05, versus EPC-NEXP group. &*P <* 0.05, versus MSC-MICE group

Est,L was significantly increased in CLP mice at days 1 and 3 compared to sham-operated animals (*P <* 0.01). Est,L was reduced significantly in the EPC-EXP and MSC-HUMAN groups compared to CLP. The EPC-NEXP and MSC-MICE groups showed no significant difference from CLP. MSC-HUMAN animals exhibited lower Est,L compared to the EPC-NEXP and MSC-MICE groups (Fig. 1).

### Expanded EPCs reduced the DAD score

**Fig. 2:**
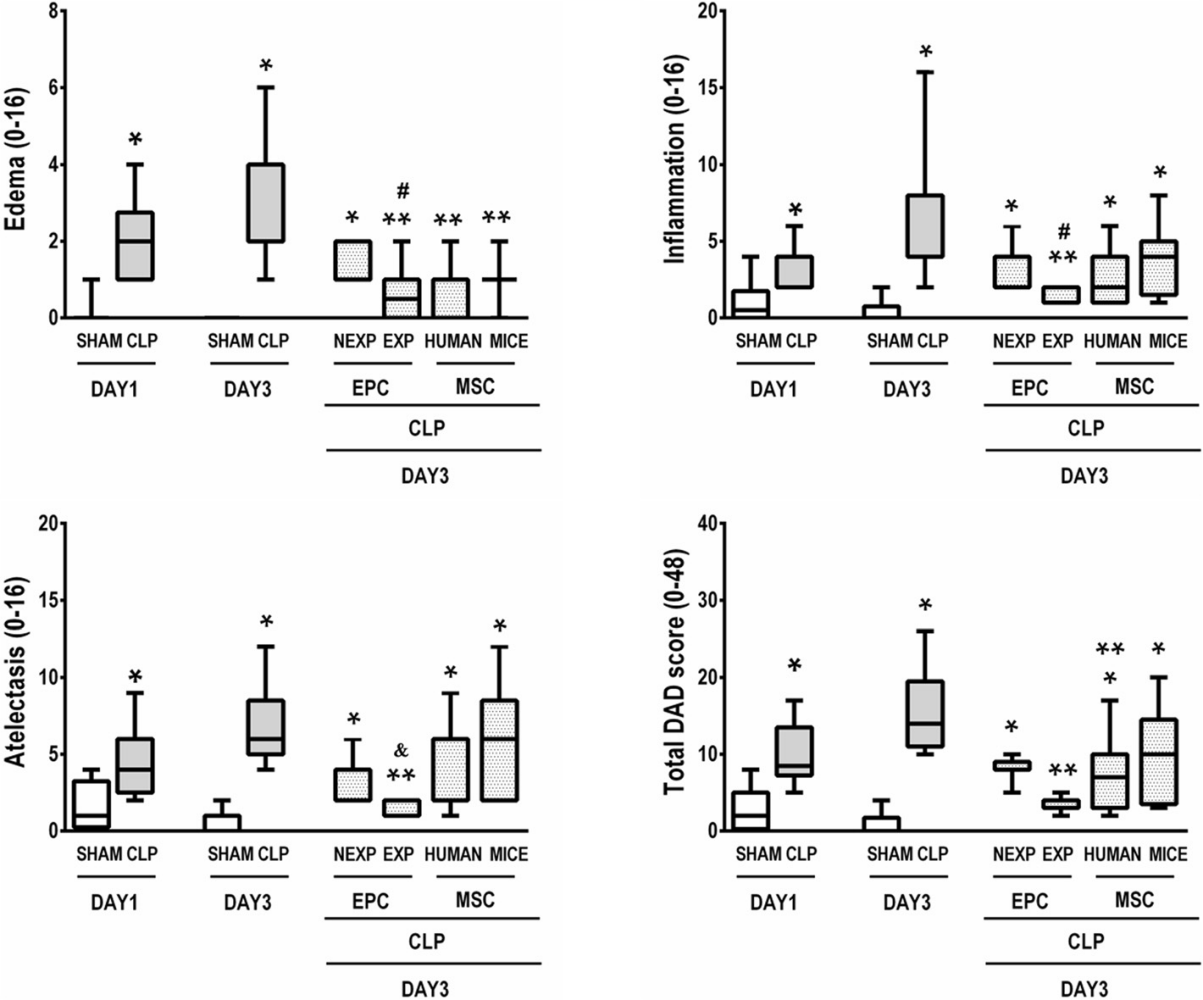
Diffuse alveolar damage in animals with lung injury induced by sepsis. Cecal ligation and puncture (*CLP*) animals were randomized to receive saline or non-expanded endothelial progenitor cells (*EPC-NEXP*), expanded endothelial progenitor cells (*EPC-EXP*), human mesenchymal stem cells (*MSC-HUMAN*), or mouse mesenchymal stem cells (*MSC-MICE*), intravenously. Values expressed as a box-and-whiskers plot of eight animals in each group. *P < 0.05, versus respective sham group. **P < 0.05, versus CLP group at day 3. ^#^ *P <* 0.05, versus EPC-NEXP group. &*P <* 0.05, versus MSC-MICE group. *DAD* Diffuse alveolar damage

Histological evaluation revealed greater edema, neutrophil infiltration, atelectasis, and total DAD score in CLP compared to sham animals at days 1 and 3. The total DAD score was reduced after EPC-EXP and MSC-HUMAN therapies compared to CLP; however, animals in the MSC-HUMAN group had higher total DAD scores than sham-operated animals. Edema was significantly decreased in the EPC-EXP, MSC-HUMAN and MSC-MICE groups compared to CLP. Inflammation and atelectasis were significantly reduced in EPC-EXP compared to CLP (Fig. 2). EPC-EXP led to reduced edema and inflammation compared to EPC-NEXP. EPC-EXP yielded decreased atelectasis compared to MSC-MICE.

### Effects of different cell therapies on inflammatory mediators and growth factors in lung tissue

**Fig. 3:**
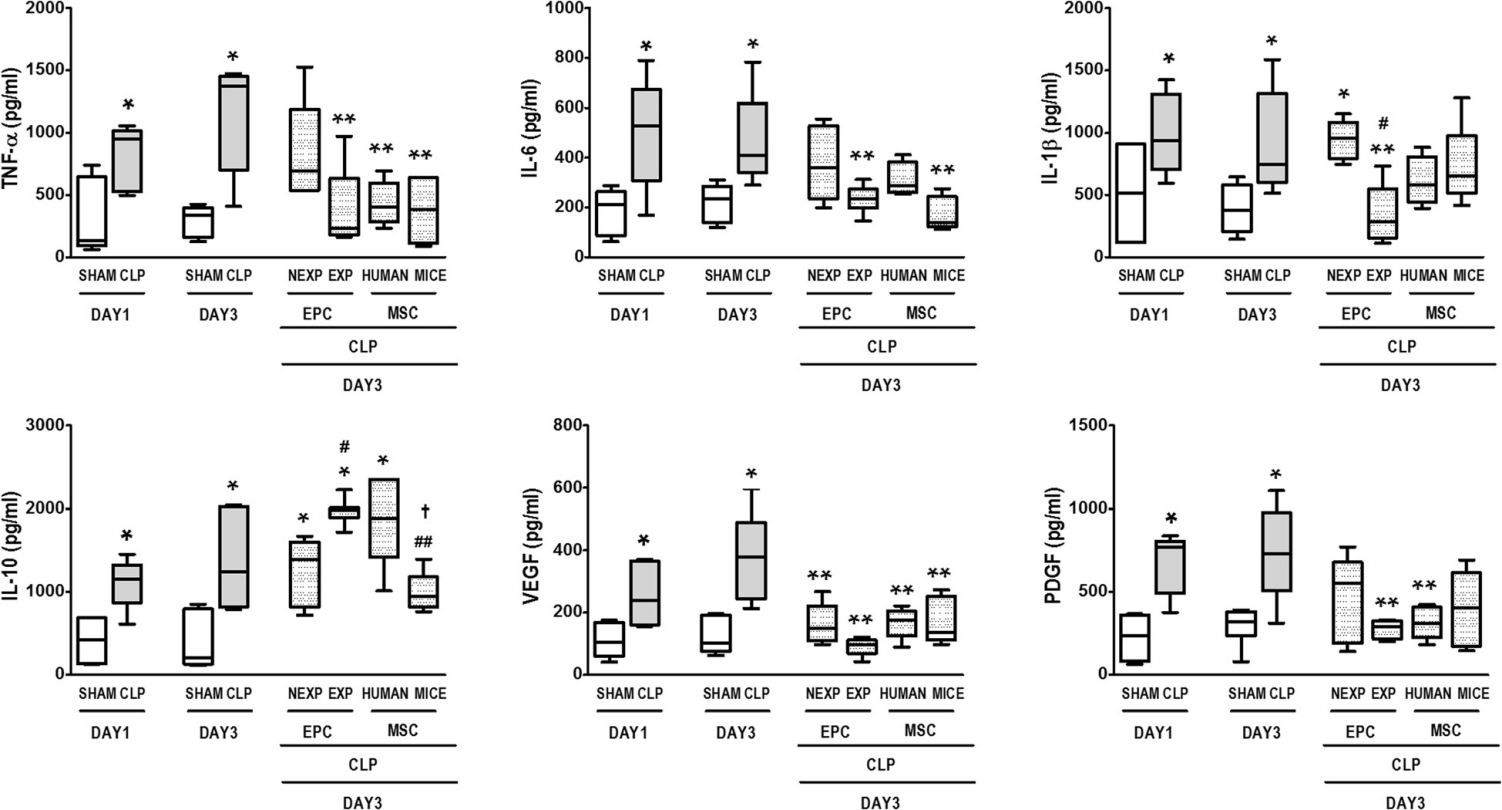
Lung inflammation on days 1 and 3. Lung tissue protein expressions of tumor necrosis factor (*TNF*)-α, interleukin (*IL*)-1β, IL-6, IL-10, vascular endothelial growth factor (*VEGF*), and platelet-derived growth factor (*PDGF*). Cecal ligation and puncture (*CLP*) animals were randomized to receive saline or non-expanded endothelial progenitor cells *(EPC-NEXP*), expanded endothelial progenitor cells (*EPC-EXP*), human mesenchymal stem cells (*MSC-HUMAN*), or mouse mesenchymal stem cells (*MSC-MICE*), intravenously. Values expressed as a box-and-whiskers plot of eight animals in each group. *P < 0.05, versus respective sham group. **P < 0.05, versus CLP group at day 3. ^#^ *P <* 0.05, versus EPC-NEXP group. ^##^ *P <* 0.05, versus EPC-EXP group. ^†^ *P <* 0.05, versus MSC-HUMAN group

Levels of inflammatory mediators and growth factors in lung tissue were higher in CLP compared to sham animals at days 1 and 3 (Fig. 3). TNF-α levels were decreased after cell therapies in the EPC-EXP, MSC-HUMAN, and MSC-MICE groups. IL-1β levels were decreased only by expanded EPC therapy, while IL-10 was decreased only by mouse MSC therapy. IL-6 level was decreased both after expanded EPC and after mouse MSC therapies. VEGF levels were decreased by all cell therapies. PDGF was decreased by expanded EPC and human MSC treatments (Fig. 3).

## Discussion

In the present study we observed that exogenously administered expanded human cord blood-derived CD133+ cells (EPC-EXP) and MSC-HUMAN were effective in improving lung morpho-function compared to CLP mice treated with saline.

Disruption of the vascular barrier is a critical step in the development of multiple organ failure in sepsis [26]. Several studies have demonstrated the role of circulating EPCs in sepsis [16, 27]. Some demonstrated that septic patients have increased numbers of circulating EPCs as compared with control subjects [16, 17]; however, another study indicated that patients with sepsis have significantly reduced numbers of circulating EPCs [27]. A recent experimental study demonstrated that mice subjected to CLP-induced sepsis had reduced circulating EPC counts at 24 hours, and that exogenous EPC administration improved survival [20].

The protective effect of expanded EPCs observed in this study is consistent with the beneficial effects of MSCs in sepsis [3, 20]. Activated MSCs could reprogram macrophages, resulting in reduced TNF-α and IL-6 but increased IL-10 production [4], which is in accordance with our results with expanded EPC administration. The EPC-EXP group experienced greater improvement of lung function and reduction of lung inflammation, whereas MSC-MICE animals exhibited reduced lung inflammation. Human MSCs also led to lung function recovery, while reducing levels only of TNF-α. One interesting finding was that IL-1β expression decreased only after EPC-EXP administration, which could explain the better overall results achieved with this therapy. IL-1β mediates inflammatory and proliferative effects in many experimental models of lung injury, including sepsis, ventilator-induced lung injury, and bleomycin [28–30]. Increased levels of IL-1β are found in the bronchoalveolar lavage fluid and serum of patients with ARDS [31, 32].

The endothelium plays an important role in sepsis, and the clinical outcome of septic patients is largely dependent on their ability to reconstitute damaged endothelium. Angiogenic factors, including VEGF signaling pathways, have recently been receiving great attention in critically ill patients, including those with sepsis [33], because of their pivotal roles in both angiogenesis and microvascular permeability. In our study, we observed a decrease in VEGF expression levels regardless of the cell therapy administered. Additionally, VEGF plays an important role in mobilizing EPCs under pathologic conditions such as cancer and sepsis [34]. While the decrease in circulating EPCs observed after anti-VEGF treatment is beneficial in cancer, it may not be so in sepsis, which may explain why expanded EPCs were effective in CLP-induced sepsis in the present study.

Several studies have shown that PDGF can accelerate tissue repair and wound healing in acute injury and in some forms of chronic injury, such as radiation-induced chronic non-healing wounds [35, 36]. Nevertheless, it is unclear whether PDGF has beneficial effects in acute critical conditions such as sepsis. Our results demonstrated that administration of expanded EPCs decreased PDGF expression levels. In a rodent model of traumatic hemorrhagic shock, administration of exogenous PDGF improved animal survival and increased tissue blood flow and mitochondrial function in vital organs [37]. However, our results demonstrated that EPC-EXP reduced PDGF expression levels, which may be explained by the different experimental models used in the aforementioned study by Liu et al. [37], in accordance with previous work published by our group with cell therapy and endotoxemia [1].

A recent study evaluated the efficacy of expanded and non-expanded EPCs in modulating myocardial function [22]. The authors observed that both expanded and non-expanded EPCs improved cardiac function. However, in our model of sepsis, in contrast to the morphofunctional benefits of EPC-EXP, EPC-NEXP did not improve lung function or histology. The reasons whereby expanded EPCs led to better lung morphofunction and reduced inflammation as compared with non-expanded EPCs remain to be elucidated, but may be associated with differences in the immunophenotype of these cells. EPC-NEXP exhibited immunophenotypic markers associated with immature cells able to differentiate into both hematopoietic and endothelial cells, depending on stimuli [20]. In contrast, EPC-EXP were already committed to the endothelial lineage and able to promote rapid neovascularization in ischemic areas immediately after infusion, thus improving the environment with nutrients and engraftment of stem cells that may act both on immunomodulation and repair of damaged tissues [20, 21]. Since these cells were studied during a short period, we may hypothesize that the mechanisms of action of EPC-EXP could be attributable to an immunomodulatory effect rather than to engraftment.

## Conclusions

In septic mice, expanded cord blood-derived EPCs and human MSCs were associated with specific improvement in lung function and histology, while the other cellular types analyzed, MSC-MICE and EPC-NEXP, were not so effective. Further studies are needed to better understand the therapeutic potential of EPC-EXP, especially as a novel therapy for sepsis.

## Additional file

Additional file 1:
**Survival rate in untreated CLP animals and those treated with cells.** Survival rate at days 1 and 3 are related to day 0; values in parentheses are survival rate related to day 1. (DOCX 12 kb)

## Abbreviations

ARDS: Acute respiratory distress syndrome
CLP: Cecal ligation and puncture
DAD: Diffuse alveolar damage
EC: Endothelial cell
EPC: Endothelial progenitor cell
EPC-EXP: expanded endothelial progenitor cell
EPC-NEXP: non-expanded endothelial progenitor cell
Est,L: Lung static elastance
IL: Interleukin
MSC: Mesenchymal stem cell
MSC-HUMAN: Mesenchymal stem cell of human origin
MSC-MICE: Mesenchymal stem cell of mouse origin
PDGF: Platelet-derived growth factor
TNF: Tumor necrosis factor
VEGF: Vascular endothelial growth factor
