# Supplementary material for: Expanded endothelial progenitor cells mitigate lung injury in septic mice
Source: Stem Cell Res Ther. 2015 Nov 26;6:230. doi: 10.1186/s13287-015-0226-7 (PMC4660838; doi:10.1186/s13287-015-0226-7)
Supplement: Additional file 1: — Survival rate in untreated CLP animals and those treated with cells. Survival rate at days 1 and 3 are related to day 0; values in parentheses are survival rate related to day 1. (DOCX 12 kb) [file 13287_2015_226_MOESM1_ESM.docx]

|  | **Day 0** | **Day 1** | **Day 3** |
| --- | --- | --- | --- |
| **CLP** | | | |
| Number of Animals | 29 | 19 | 17 |
| Survival Rate |  | 65.5% | 58.6 % (89%) |
| **MSC-MICE** | | | |
| Number of Animals | 35 | 23 | 22 |
| Survival Rate |  | 65.7% | 62.9 (95.7%) |
| **MSC-HUMAN** | | | |
| Number of Animals | 32 | 22 | 18 |
| Survival Rate |  | 68.8% | 56.3% (81.8%) |
| **EPC-NEXP** | | | |
| Number of Animals | 31 | 21 | 16 |
| Survival Rate |  | 67.7% | 51.6% (76.2%) |
| **EPC-EXP** | | | |
| Number of Animals | 26 | 17 | 17 |
| Survival Rate |  | 65.4% | 65.4% (100%) |

Additional File 1. Survival rate in untreated CLP animals and those treated with cells

Survival rate at days 1 and 3 related to day 0. Values in parenthesis (survival rate related to day 1).
